# Local Intracoronary Eptifibatide versus Mechanical Aspiration in Patients with Acute ST-Elevation Myocardial Infarction

**DOI:** 10.1155/2014/294065

**Published:** 2014-06-03

**Authors:** Mohamed A. Hamza, Ayman Galal, Salwa Suweilam, Mohamed Ismail

**Affiliations:** ^1^Ain Shams University, P.O. Box 11381, Cairo, Egypt; ^2^Cardiology Department, Ain Shams University, Lotfy Elsayed Street, Abbaseya, P.O. Box 11381, Cairo, Egypt

## Abstract

*Objectives*. We compared local delivery of intracoronary eptifibatide via perfusion catheter to thrombus aspiration in primary PCI. *Background*. Perfusion catheter increases local concentration of the drugs at the culprit site and prolongs their residency time. *Methods*. 75 patients with acute STEMI were randomized to three groups: 25 received local intracoronary eptifibatide and verapamil via perfusion catheter; 25 patients were managed by Diver CE thrombectomy device and 25 patients by primary PCI without thrombus aspiration. Primary end point was assessment of postprocedural TIMI flow, MPG, and corrected TIMI frame count (cTFC) in the culprit vessel. *Results*. Perfusion catheter was superior to thrombus aspiration and conventional PCI as regards MBG (68% versus 36% in Diver CE and 20% in the control arm; *P* value = 0.002), with shorter cTFC rates than thrombectomy and control groups (20.76 ± 4.44 versus 26.68 ± 8.40 and 28.16 ± 5.96, resp.; *P* = 0.001). TIMI flow was not different between the 3 groups. Eptifibatide led to less time to peak CK (13.12 hours versus 16.5 and 19.5 hours, respectively, *P* value = 0.001). *Conclusion*. Local intracoronary eptifibatide by perfusion catheter reduces thrombus burden with better results in microvascular perfusion assessed by cTFC and MBG compared to aspiration device or conventional PCI.

## 1. Introduction

Primary percutaneous coronary intervention (PCI) is considered the preferred reperfusion modality for patients presenting with ST-segment elevation myocardial infarction (STEMI) regardless of the hour of presentation as long as reperfusion can occur in a timely manner [1].

Yet despite the prompt and successful restoration of ante grade epicardial blood flow by PCI, a significant proportion of patients with AMI remain at increased risk of death and adverse outcomes [2]. Also microvascular obstruction with diminished myocardial perfusion occurs in a large proportion of patients with a patent epicardial vessel after primary PCI, and this event is associated with an increased infarct size, reduced recovery of ventricular function, and increased mortality [3].

The high frequency of suboptimal myocardial reperfusion after primary PCI has resulted in the development of different feasible and safe thrombectomy and distal protection devices. The efficiency and safety of adjunct thrombectomy using Diver CE device (Invatec, Italy) have been established [4].

A promising novel solution to ameliorate the outcome of angioplasty for acute coronary syndromes resides in the combined use of pharmacological and catheter-based therapies to increase local concentration of drugs such as glycoprotein IIb/IIIa inhibitors at the culprit site, prolonging their residency time [5].

The therapeutic perfusion catheter is a microporous balloon catheter that acts as a low-pressure irrigating system for localized perfusion of therapeutic agents into the coronary vasculature providing up to 500 times the systemic concentration of a drug by gently occluding the blood flow and significantly increasing the residence time of the infused drug at the specific target site [6].

We investigated the efficacy of local eptifibatide delivery to the site of thrombus through infusion catheter in comparison with thrombus aspiration in patients with acute STEMI undergoing primary PCI.

## 2. Methods

### 2.1. Study Design and Patient Selection

The study population consisted of 75 patients who presented to Ain Shams University hospital with ST-elevation myocardial infarction (STEMI) and planned for primary PCI; they were randomized to management either by local intracoronary eptifibatide through infusion catheter (*n* = 25) or by aspiration thrombectomy device (*n* = 25) or managed by conventional PCI (*n* = 25).

The following was excluded from the study:

1. use of a fibrinolytic agent within 14 days before PCI,
2. suspected active internal bleeding,
3. history of cerebrovascular accident within the previous 2 years,
4. known platelet count <100,000 cells/*μ*L.

Eptifibatide was the GP IIb/IIIa used as it is more cost effective and frequently used in our country. Patients were randomly assigned to receive local delivery of 180 *μ*g/kg intracoronary eptifibatide given via infusion catheter (then intravenous maintenance dose, 2.0 *μ*g/kg-min, for 12 hr) plus 100 *μ*g Isoptin or thrombus aspiration using Diver CE catheter, introduced in a guiding catheter 6f (>0.068 inches internal diameter) or conventional PCI with or without PTCA.

The three arms were treated finally by stenting of the culprit lesion. All patients received weight adjusted dose of heparin during the procedure, aspirin 300 mg orally as soon as possible and 150 mg daily thereafter, a clopidogrel 600 mg loading dose before PCI, and 75 mg daily for at least 3 months after randomization (or up to 1 year in case of drug-eluting stent implantation), statins beta blockers, and ACE inhibitors.

Laboratory investigations were done for all patients including serum creatinine level, CBC, and troponin on admission and 6 hours after and also CK-MB every 6 hours till normalization. Echocardiography was done during hospital stay to all patients to measure EF using Simpson's method.

### 2.2. End Points

The primary end points were postprocedural assessment of TIMI flow, corrected thrombolysis in myocardial infarction (TIMI) frame count, and myocardial blush grade [7]; these parameters were measured by the PCI operator who was blinded. The secondary end point was procedure-related myocardial infarction (MI) and in-hospital stay rates of major adverse cardiac events (MACE). MACE was defined as the composite of death from any cause, reinfarction, or target vessel revascularization. Procedure-related MI was diagnosed if the creatine kinase-myocardial band (CK-MB) level increased to be twice the last nonnormalized measurement [8].

TIMI thrombus grading classification was used to evaluate thrombus burden; patient was considered to have angiographically evident thrombus if TIMI thrombus grades 2 to 5 were present [9].

### 2.3. Statistical Analysis

The collected data were coded, tabulated, and statistically analyzed using the SPSS program (Statistical Package for Social Sciences) software version 17.0.

Descriptive statistics were done for numerical parametric data as mean ± SD (standard deviation) and minimum and maximum of the range, while they were done for categorical data as number and percentage.

Inferential analyses were done for quantitative variables using independent *t*-test in cases of two independent groups with parametric data and one way ANOVA for two or more means. Inferential analyses were done for qualitative data using Chi square test. Pearson correlation was used to measure the correlation (linear dependence) between two variables. The level of significance was taken as follows: *P* value < 0.05 is significant; otherwise it is nonsignificant. The *P* value is a statistical measure for the probability that the results observed in a study could have occurred by chance.

## 3. Results

### 3.1. Study Population

During the study period, 75 patients who presented to Ain Shams University hospital with ST-elevation myocardial infarction (STEMI) and planned for primary PCI were randomized to management either by local intracoronary eptifibatide through perfusion catheter (*n* = 25) or by aspiration thrombectomy device (*n* = 25) or managed by conventional PCI (*n* = 25). Baseline demographic data, including age, gender, cardiovascular risk factors, and infarct related vessel, is presented in Table 1.

In all our study population the duration of chest pain (pain-to-door) had mean value of 5.18 hours (SD ± 3.96), while the door-to-balloon time had mean value of 43.85 minutes (SD ± 20.6) ranging from 15 to 100 minutes.

### 3.2. Parameters of Success of Reperfusion

In the infusion catheter group, the peak CK-MB reached had mean value of 216.88 ± 75.46, versus 368.60 ± 217.02 in the thrombus aspiration group and 351.72 ± 217.02 in the control group. Post hoc analysis showed that the control group and the thrombus aspiration group were both significantly higher in CK-MB level compared to perfusion catheter group (*P* value = 0.004, *P* value = 0.011, resp.).

The mean time to reach peak of CK was 13.12 ± 4.25 hours in the local intracoronary infusion group, 16.56 ± 5.43 hours in the thrombus aspiration group, and 19.52 ± 6.44 hours in the control group. Which is significantly lesser in the perfusion catheter group compared to both aspiration group and control group (*P* value = 0.015, *P* value = 0.001, resp.)

In the local eptifibatide group the magnitude of ST resolution had mean value of 56.88% (SD 15.14), while in the aspiration group it was 59.6% (SD 21.5) and 51.52% (SD 26.28) in the control group (*P* value = 0.404). Also in the infusion catheter group the mean ejection fraction measured after infarction was 46.64 ± 6.66%, in the thrombus aspiration group was 41.76 ± 8.38%, and in the control group was 41.88 ± 9.85% (*P* value 0.071).

### 3.3. Myocardial Reperfusion

**Figure 1:**
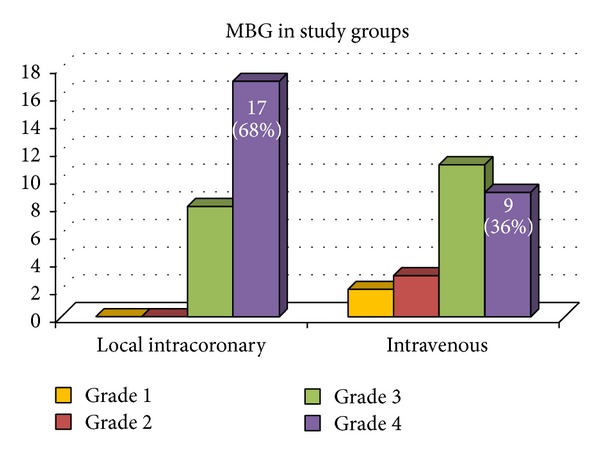
Comparison between 3 groups in MBG.

In the infusion catheter group 68% of patients had MBG 3 compared to 36% of patients in thrombus aspiration group and 20% of patients in the control group, with proven significant increase in the number of patients with MBG 3 in infusion catheter group as compared to the other 2 groups (*P* value = 0.002). Also the infusion had cTFC shorter than the aspiration and control group (20.76 ± 4.44 versus 26.68 ± 8.40 and 28.16 ± 5.96), respectively (*P* value = 0.001) (Figure 1).

There was no significant increase in the number of patients with TIMI 3 flow in the infusion catheter group (84% versus 80% of patients in both thrombus aspiration and control groups) (*P* = 0.916). Also there were no recorded clinical events during hospital stay in three groups.

## 4. Discussion

To the best of our knowledge, this is the first study comparing local delivery of intracoronary eptifibatide using infusion catheter to the use of thrombus aspiration in primary PCI in patients with acute STEMI. We started this work to evaluate the newly introduced device (perfusion catheter) regarding its efficacy in management of thrombus containing lesion.

Intracoronary eptifibatide is associated with improved microvascular perfusion demonstrated by an improved cTFC in the ICE trial [10]. The presence and lowering WMI of high localized concentration of drug may permit the dissociation of the bound fibrinogen with platelets to form the occlusive thrombus. Hence microvascular perfusion may be improved by reducing both the number and size of microemboli. This mechanism was seen in in vitro studies modeling coronary flow [11, 12]. Further more recent studies have demonstrated that higher concentrations of GP IIb/IIIa receptor antagonists are necessary to effectively disaggregate stable and aged aggregates compared with newly formed thrombi [13]. Also, Intracoronary verapamil injection is beneficial in preventing no-reflow/slow-flow, reducing cTFC, and improving MPG [14].

The current study showed that intracoronary eptifibatide was significantly superior to using thrombus aspiration catheter and to conventional PCI; as regards myocardial reperfusion expressed by MBG, 68% of patients in the local drug delivery arm had MBG 3 versus 36% and 20% of patients in the other groups, respectively. The CRYSTAL AMI study (a pilot study before INFUSE AMI) shows similar results, while COCTAIL study [15] found a nonsignificant increase in MBG with use of infusion catheter, and also the INFUSE AMI [16] trial did not find significant difference between its groups as regards MBG.

CRYSTAL AMI, which compared IV abciximab and intracoronary abciximab in 50 patients with STEMI, showed that intracoronary abciximab catheter led to better myocardial perfusion as shown by higher number of patients with MBG 3 score compared to IV abciximab (75% versus 45%, resp.). The COCTAIL study, which randomized 50 patients with non-ST segment elevation myocardial infarction to administration of abciximab by local intracoronary infusion versus intracoronary infusion through guiding catheter, found a nonsignificant increase in MBG compared to IC abciximab infusion through guiding catheter.

The INFUSE AMI evaluated the use of intracoronary abciximab and aspiration thrombectomy (export catheter) in 452 patients undergoing primary PCI for anterior STEMI and did not find significant difference between IC abciximab and the other arm as regards MBG scores. This finding may be related to the structure of the INFUSE AMI trial itself, where part of the group of patients who did not receive IC abciximab underwent thrombus aspiration by export, hence decreasing thrombus load and improving MBG scores. In addition one limitation concerning INFUSE AMI trial was that all patients had only anterior STEMI.

In the present study postprocedural TIMI flow did not reach statistical significance; this is similar to results of COCTAIL and CRYSTAL AMI studies. Also, our study showed that the intracoronary eptifibatide group had better myocardial perfusion results assessed by significantly less cTFC rates than thrombus aspiration and control groups (20.76 ± 4.44 versus 26.68 ± 8.40 and 28.16 ± 5.96, resp.). These results are concordant with the results of COCTAIL study, while the INFUSE AMI trial did not find significant effect for using perfusion catheter on cTFC rates after procedure. The COCTAIL study illustrated significantly lower cTFC rates in group with intracoronary abciximab via infusion catheter compared to group with intracoronary abciximab delivered via guiding catheter (15.3 ± 10.2 versus 21.1 ± 9.9, *P* = 0.049).

The results obtained by this study showed a good safety profile of the infusion catheters with promising results that may improve clinical outcome and decrease thrombus burden and complications related to microvascular occlusion.

*Limitations.* The small number of the patients is the main limitation of this study as is it was not large enough to increase the power of our results; however this field is really interesting and further larger scaled studies are needed to prove or disprove the results in this study.

## Figures and Tables

**Table 1:** Baseline demographic data in the three groups.

	Perfusion catheter	Thrombus aspiration	Conventional PCI	*P* value
Gender				
Male	20 (80)	22 (88)	24 (96)	0.220
Female	5 (20)	3 (12)	1 (4)	
Age	49.52 ± 9.62	53.72 ± 9.33	56.24 ± 11.45	0.069

Smoking	21 (84)	20 (80)	19 (76)	0.779
Dyslipidemia	12 (48)	12 (48)	15 (60)	0.618
DM	9 (36)	8 (32)	11 (44)	0.671
HTN	8 (32)	9 (36)	10 (40)	0.841
FH	6 (24)	2 (8)	2 (8)	0.158

Vessel related infarction				
LAD	12 (48)	15 (60)	16 (64)	
RCA	12 (48)	10 (40)	9 (36)	
LCX	1 (4)	0	0	
